# Insight into MAS: A Molecular Tool for Development of Stress Resistant and Quality of Rice through Gene Stacking

**DOI:** 10.3389/fpls.2017.00985

**Published:** 2017-06-13

**Authors:** Gitishree Das, Jayanta Kumar Patra, Kwang-Hyun Baek

**Affiliations:** ^1^Research Institute of Biotechnology and Medical Converged Science, Dongguk University SeoulGoyang-si, South Korea; ^2^Department of Biotechnology, Yeungnam UniversityGyeongsan, South Korea

**Keywords:** gene pyramiding, genome mapping, phenotype traits, physiological traits, molecular markers, marker assisted selection, rice

## Abstract

Rice yield is subjected to severe losses due to adverse effect of a number of stress factors. The most effective method of controlling reduced crop production is utilization of host resistance. Recent technological advances have led to the improvement of DNA based molecular markers closely linked to genes or QTLs in rice chromosome that bestow tolerance to various types of abiotic stresses and resistance to biotic stress factors. Transfer of several genes with potential characteristics into a single genotype is possible through the process of marker assisted selection (MAS), which can quicken the advancement of tolerant/resistant cultivars in the lowest number of generations with the utmost precision through the process of gene pyramiding. Overall, this review presented various types of molecular tools including MAS that can be reasonable and environmental friendly approach for the improvement of abiotic and biotic stress resistant rice with enhanced quality.

## Introduction

Rice, which is the world's most important food crop, is a key source of carbohydrate (Narciso and Hossain, 2002). The cultivation of rice is a principal activity and source of income around the world, and several Asian and African countries depend on rice as a basis of earnings (Khan et al., 2009). However, there is severe yield loss in rice cultivation due to a number of abiotic and biotic stresses (Ramegowda and Senthil-Kumar, 2015). Several environmental factors have threatened sustainable agricultural production in emerging countries, with the main variables affecting the future of agricultural production being higher incidence of extreme weather and a number of environmental problems (Joneydi, 2012).

Biotic stresses including pathogens, pests and weeds and abiotic stresses such as drought and periodic cycles of submergence, extreme cold, soil salinity, affects rice cultivation throughout the world. Crop losses caused by major biotic stressors such as bacterial blight and blast disease, and due to insect pests are quite high (Hasan et al., 2015). The occurrence of new stresses necessitates development of highly improved and novel approaches to enhance the capability of various rice varieties that can survive attacks caused by several pathogens at once while also surviving in unfavorable environments with high level of grain quality. In conventional breeding techniques, along with the desired genes other unwanted genes also continue to a long term in next generations, even with several backcross generations and it is not possible to screen unwanted genes, using conventional breeding. Despite their limitations, conventional approaches are also important for conserving wild germplasms, sexual hybridization between contrasting parental lines, novel genetic variants, and mutations (Werner et al., 2005). A variety of methods are used in conventional breeding, such as backcrossing, recurrent selection, and mutation breeding methods. However, using molecular tools such as markers that flank a target gene, can minimize the number of backcross generations (Hasan et al., 2015).

Molecular marker techniques are currently the most advanced method available for the transfer of desired gene in desired rice variety with required combination. The improvement of breeding programs using the most widely used molecular techniques, and their application is a novel prospect for enhancement of rice yields. Hence, the present study was conducted to briefly review the adverse effects of various types of biotic and abiotic stresses on production of rice and to improve the resistance with higher grain quality of rice through application of various types molecular tools especially the MAS.

## Marker assisted selection (MAS): an advanced molecular tool in rice breeding

MAS is a process in which a marker is used for indirect selection of a genetic determinant or determinants of a trait of interest, i.e., abiotic stress tolerance, disease resistance, productivity, and/or quality (Prabhu et al., 2009). This method involves selection of plants carrying genomic regions that are involved in the expression of traits of interest through the application of molecular markers. The development and availability of an array of molecular markers and dense molecular genetic maps in crop plants has made application of MAS possible for traits governed by major genes and QTLs (Choudhary et al., 2008). The success of MAS depends on several factors, including the number of target genes to be transferred and the distance between the flanking markers and the target gene (Perumalsamy et al., 2010). MAS is gaining considerable importance as it can improve the efficiency of plant breeding through precise transfer of genomic regions of interest and acceleration of the recovery of the recurrent parent genome (Wijerathna, 2015).

### Marker and QTL identification for MAS

The identification of DNA markers, genes and quantitative trait loci (QTLs) associated with particular traits is accomplished through QTL mapping. As a result, QTL mapping signifies the basis of development of molecular markers for MAS. However, there are several aspects that affect the accuracy of QTL mapping, such as replication levels of phenotypic information, population sizes and types, genotyping errors, and environmental effects. Genes or QTLs can be detected relative to a linkage map, by means of statistical methods such as single-marker analysis or interval mapping to identify associations between DNA markers and phenotypic data (Kearsey and Farquhar, 1998). Using DNA markers for identification of QTLs was a breakthrough in the characterization of quantitative traits. In plants, the identification of genomic regions related to quantitative traits has mostly been attained through QTL mapping (Borba et al., 2010).

### Advantages of MAS for the improvement of stress resistant rice

With the application of MAS, individual plants can be selected based on their genotype during the selection procedure. For most traits, homozygous and heterozygous plants cannot be distinguished by conventional phenotypic screening. MAS can be used to assist selection of parents, increasing the effectiveness of backcross breeding and improving sex-limited traits (Zhou et al., 2007). MAS can be used to investigate heterosis for hybrid crop production (Reif et al., 2003), and there is the potential for use of DNA marker data along with phenotypic data to select hybrids (Jordan et al., 2003).

**Figure 1:**
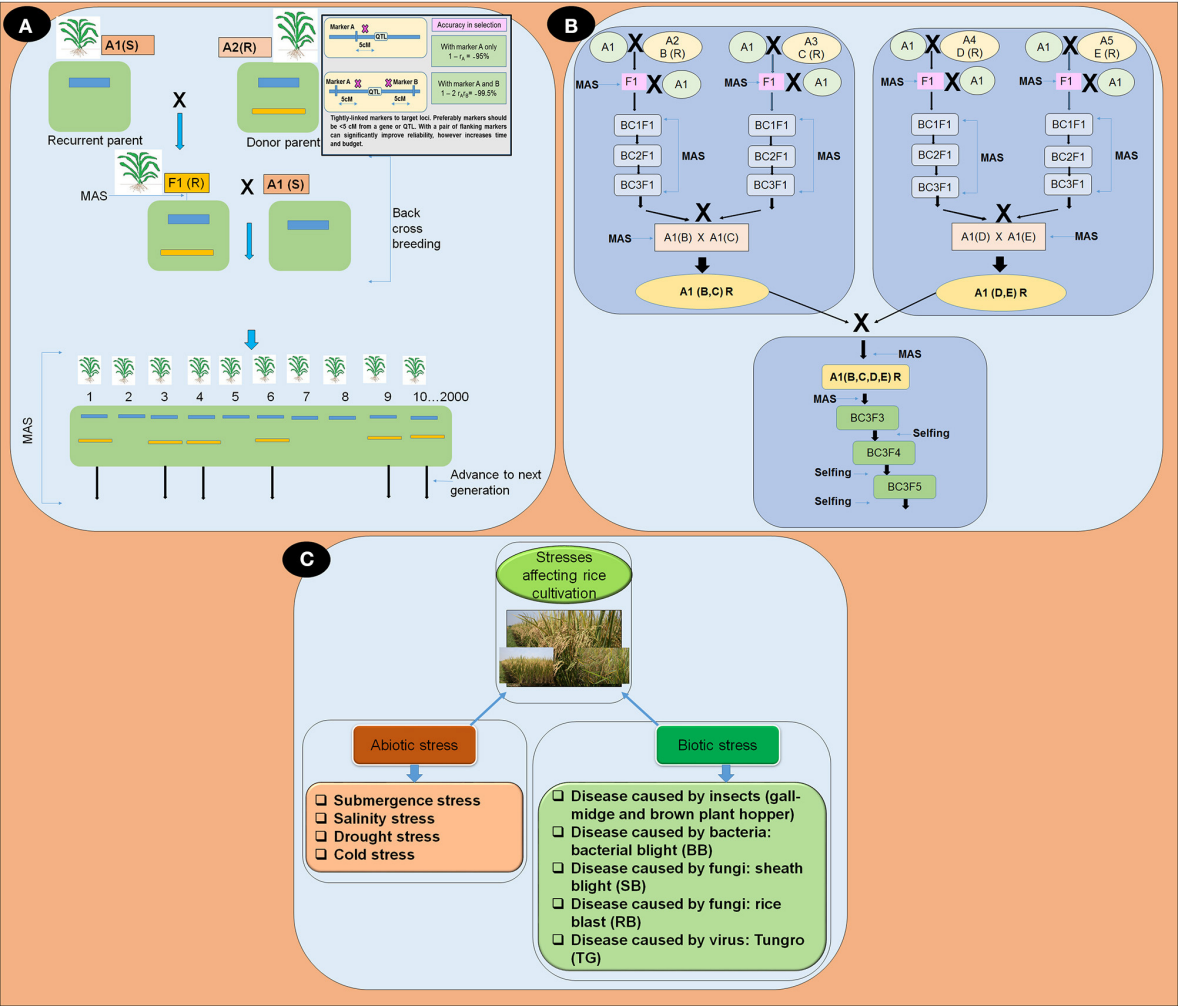
**(A)** Overview of marker assisted backcross breeding program; **(B)** Flow diagram depicting the gene pyramiding of multiple stress resistance (R) genes into a single line using marker assisted backcross breeding; and **(C)** Flow diagram of stresses affecting rice productivity.

There are various advantages of using MAS in rice breeding. For example, it may be simpler than phenotypic screening; therefore, it can reduce time, effort, and resources. Selection of quality traits in rice generally requires expensive screening procedures that are made feasible through MAS (Figure 1A). Additionally, MAS selection can be conducted at the seedling stage and undesirable plant genotypes can quickly be eliminated (Khan et al., 2015). The advantages associated with the use of markers includes speed, consistency, efficiency, biosafety, and the ability to skew the odds in our favor, even while dealing with complex traits.

## Molecular markers used in rice breeding

The development of novel molecular tools has significantly influenced rice breeding programs, permitting application of advanced molecular breeding techniques in rice that leads to the improvement of novel rice varieties through advanced biotechnology, which majorly includes MAS and genetic engineering.

Several researches shown that, in plants, single-nucleotide polymorphisms (SNPs) in addition to insertions and deletions (InDels) are extremely abundant and distributed throughout the genome (Batley et al., 2003). In plant genomes the abundance of polymorphisms makes the SNP marker system a smart tool for marker-assisted selection in breeding program (Hayashi et al., 2004). 1.7 million SNPs have been identified in rice, by comparative analysis of the draft genomic sequences of cv. *Nipponbare* (Kurokawa et al., 2016). Using NGS (Next-generation sequencing) technologies transcriptome resequencing permits fast and reasonable SNP detection within genes and avoids extremely repetitive sections of a genome (Mammadov et al., 2012). In the rice genome, SNPs are the most frequent polymorphism. In recent times, SNP arrays have been developed for rice by breeders, which are designed to cover a wide range of cross combinations in rice. For separating “linkage drag” the tightly linked set of SNPs can be used. The SNP array can also be readily used to construct an NIL (Near isogenic line) with a very minor introgressed chromosome segment from the donor parent, if a large segregating population is existing (Kurokawa et al., 2016). The employment of SNPs as molecular markers for breeding is becoming an actual possibility.

Advancement of molecular techniques in rice breeding has significantly extended the applicability of identification of genes and pyramiding valuable genes (Wijerathna, 2015). In gene pyramiding for a variety MAS not only shortened the breeding period but also removed the extensive trait assessment involved. There are many rice varieties improved by MAS (Table 1) (Rao et al., 2014). The steps of gene pyramiding using marker assisted selection (MAS) is displayed in (Figures 1A,B). There are many stress resistance genes and genes related to quality of rice which are tightly linked SNPs, SSRs, and STS markers are available (Table 1). All DNA marker techniques are designed to detect and exploit naturally occurring DNA polymorphisms. The main considerations for the use of DNA markers in MAS are as follows: particular markers should be tightly linked to target loci, preferably less than 5 cM genetic distance (Figure 1A).

**Table 1:** Selected lists of genotypes improved by MAS; selected abiotic and biotic stress resistance genes/QTLs and linked markers; selected stresses, resistance genes/QTLs, and their donor parents.

**List of genotypes improved by MAS**			
**Genotypes**	**Rice variety improved by MAS**	**Traits and resistant genes**	**References**
Pusa basmati I	Improved Pusa Basmati I	Bacterial blight (*xa13 + Xa21*)	Kottapalli et al., 2010
Samba Mahsuri (BPT-5204)	Improved Samba Mahsuri	Bacterial blight (*xa5 + xa13 + Xa21*)	Kottapalli et al., 2010
Swarna	Swarna sub1	Submergence (Sub1)	Nandi et al., 1997
IR64	IR64 Sub1	Submergence (Sub1)	Reddy et al., 2009
Pusa RH 10	PRR78/IRBB60	Rice blast (*Pi54* + *Piz5*)	Singh et al., 2011
Pusa RH 10	Pusa 6A, Pusa 6B	Bacterial blight (*xa13* + *Xa21*)	Singh et al., 2011
KMR3 Restorer	KMR3/IRBB60	*Xa4 + xa5* + *xa13* + *Xa21*	Shanti et al., 2010
Lalat	Improved Lalat	Bacterial blight *(Xa4 + xa5* + *xa13* + *Xa21)*	Dokku et al., 2013
Tapaswini	Improved Tapaswini	Bacterial blight *(Xa4 + xa5* + *xa13* + *Xa21)*	Dokku et al., 2013
Mangeumbye	Improved Mangeumbye	Bacterial blight (*Xa4 + xa5 + Xa21*)	Suh et al., 2013
PRR78	Improved Pusa RH10	Rice blast (*Piz5* + *Pi54*)	Singh et al., 2013
Wuyujing 3	K01, K04	Low-amylose content gene (*Wx-mq*)	Tao et al., 2016
**List of selected abiotic and biotic stress resistance and quality genes/QTLs and linked markers**			
**Stress/Disease/Traits**	**Resistance genes/QTLs**	**Available linked markers**	**References**
Submergence	*Sub1*	SUB1BC2, RM464A, RZ698, C1232, RG381 and RG345	Das and Rao, 2015
Salinity	*Saltol*	RM8094, RM140, RM10745, RM10772	Nejad et al., 2008, 2010; Das and Rao, 2015
Drought	*QTLs*	RM212, RM319, RM316, RM537	Lin et al., 2007
Cold	*qPSST-3, qPSST-7, qPSST-9, qSCT1a, qSCT2*	RM231, RM1377, RM24545, RM3602 RM1211	Jena et al., 2010; Kim et al., 2014
Heat	*qHTSF4.1*	M4	Ye et al., 2015; Nogoy et al., 2016
Gall midge	*Gm1, Gm2, Gm4*	RM444, RM316, and RM219, RG476, RG329, RM547	Biradar et al., 2004; Das and Rao, 2015
Hopper burn	*Bph-1* and *Bph-10* (t), *Bph-3, Bph-17, Bph-18, Bph-20, Bph21, Bph25, Bph26*	XNpb248 and RG457, RM589, RM5953, RM6217, BP-20-2, B121,RM6273, RM6775, RM5479	Singh et al., 2011; Kurokawa et al., 2016
BB	*Xa21, xa13, Xa10, xa5, Xa4, Xa3, Xa1, Xa7*	pTA248, AB9, RG103, Xa13p, RG136, O07_2000_,CDO365, RG556, XNpb181, R1506-S12886, MP, XNpb181, Y5212L, C600, Y5212R, 16PFXa1/EcoRV, M5	Ma et al., 1999; Porter et al., 2003; Shin et al., 2007; Singh et al., 2011; Kottearachchi, 2013; Kurokawa et al., 2016
SB (sheath blight)	*qShB1, qShB2-1, qSB5, qShB6, qShB9-2*	RM104, RM341, RM13, RM190, RM245	Liu et al., 2009
RB (rice blast)	*Pi-1, Pi 2, Pi 4(t), Pi 5, Piz-5, Pi 5(t), Pi 7 (t), Pi 10 (t), Pi-b, Pi54, Pi21, Pia*	RZ536, RG64, RG869, S04G03, AP4007, AP5930 RG498, RG788, RG103A, RG16, RRF6, RZ213, RZ123, G1234, RM206, Os04g0401000, YCA72	Cho et al., 2007; Singh et al., 2011; Kottearachchi, 2013
TG (Tungro)	RTSV *replicase gene*	RZ262	Khondker et al., 2005
Deep roots	QTLs on chromosomes 1, 2, 7 and 9	RFLP and SSR markers	Hasan et al., 2015
Root traits C Aroma	QTLs on chromosomes 2, 7, 8, 9 and 11	RFLP and SSR markers	Hasan et al., 2015
Heading date	QTLs for heading date (Hd1,Hd4, Hd5, or Hd6)	RFLP, STS, SSR, CAPS, dCAPs	Hasan et al., 2015
Quality	Waxy	RFLP	Hasan et al., 2015
Eating quality	Amylose content gene	RM190	Jairin et al., 2009
Fragrance	Fragrance gene	BO3_127.8	Jairin et al., 2009
High yield	*Gn1a/OsCKX2, APO1, WFP/OsSPL14*	Os01g0197700, Os06g0665400, Os08g0509600	Ashikari et al., 2005; Kurokawa et al., 2016
Seed shape	*GW2, GS3, qSW5*	Os02g0244100, Os03g0407400, Os05g0187500	Song et al., 2007; Shomura et al., 2008; Takano-Kai et al., 2009; Kurokawa et al., 2016
**List of selected stresses, resistance genes/QTLs and their donor parents**			
**Disease/Stress**	**Resistance genes/QTLs**	**Donor parents**	**References**
**BIOTIC STRESS**			
Bacterial blight	*Xa1, Xa2, Xa3, Xa4, Xa5, Xa10, Xa13, Xa21*	Kogyku, Tetep, Chogoku 45, IR20, IR1545-339, CAS209, *O. longistaminata*, Pusa 1460	Swamy et al., 2006; Singh et al., 2011
Rice blast	*Pi-1(t), Pi-2(t), Pi-4(t), Pi-5(t), Pi-zh, Pi2, Pi9, Piz-5, Pi54, Piz, pi21*	LAC23, 5173, Tetep, IRAT13, Moroberekan, Zhiyeqing, C1O1A51, *O. minuta derivative*, Pusa 1602, IRBLZ5-a, DHMAS-70 Q164-2a,z2143,z1671, Os04g0401000	Hayashi et al., 2004; Deng et al., 2006; Fukuoka et al., 2009; Singh et al., 2011; Kurokawa et al., 2016
Gall midge	*Gm1, Gm2, Gm4(t), Gm5(t), Gm6(t), Gm7(t), Gm8(t), Gm9(t), Gm10(t), Gm11(t)*	Kavya, Siam 29, Abhaya, ARC5984, Duokang #1, Bhumansan, NHTA 8, Banglei	Jain et al., 2004; Kumaravadivel et al., 2006; Dubey and Chandel, 2010
RTSV and GLH	*Replicase genes, Glh-1, Glh-2, Glh-3, Glh-4, Glh-5, Glh-6, Glh-7, Glh-8, Grh4, Grh2*	Taipei 309-147.4 and Taipei 309-147.8, ARC11554, Pankhari 203, ASD7, IR8, Ptb8, Tightly linked to XNpb144, Tightly linked to G1465	Khondker et al., 2005; Kurokawa et al., 2016
BPH	*Bph1, Bph2, Bph3, Bph4, Bph5, Bph6, Bph7, Bph8, Bph9, Bph10(t),Bph20(t), Bph21(t) Bph12(t), Bph13(t), Bph14 (Qbp1) and Bph15 (Qbp2)*	Mudgo, ASD7, Rathu Heenati, Babawee, ARC10550, Swarnalata, T12, Chin Saba, pokkali, *O. australiensis, Oryza latifolia, Oryza eichingeri, Oryza officinalis*	Sun et al., 2006; Yadavalli et al., 2011
**ABIOTIC STRESS**			
Drought	*Dreb1*	Nagina 22	Reddy et al., 2009
Submergence	*Sub1*	FR13A, Swarna sub1, IR64 sub1, FR43B, Kurkurappan and Thavalu	Endang et al., 2009, 2012
Salinity	*Saltol*	FL496, FL478, FL378, Pokkali, SR26B, Patnai 23, Vytilla 1	Reddy et al., 2009; Nejad et al., 2010
**QUALITY**			
Eating quality	Low-amylose content gene Wx-mq	Kanto 194	Tao et al., 2016

By exploring the diverse molecular techniques and advanced genomic technologies such as genome sequencing, SNPs array, genome-wide association mapping, and transcriptome profiling, the molecular mechanism and their relation between the genotypes and phenotypic traits leading to development of improved rice varieties can be realized (McCouch et al., 2016; Peng et al., 2016). Currently SSRs (second-generation markers) are widely used markers in MAS due to the easy availability and comparatively cheaper than others and they require a comparatively simple technique with a higher polymorphism rate (Gao et al., 2016).

### Molecular markers for phenotype and physiological traits

The QTL analysis is an essential step to phenotyping of mapping of the population for their resistance to biotic and tolerance to abiotic stress. By pyramiding resistance genes through MAS, more durable resistance can be achieved via the identification of additional QTLs. Generally, biotic stress resistance is monitored using a specific scale value. Alternative methods such as quantification of the infected area can be used or tested for it. Phenotyping for abiotic stress is also essential to reducing the gap between genotype and phenotype, particularly for quantitative traits, which are the prime factors of abiotic stress resistance (Boopathi, 2012). The future of plant breeding depends on the complete knowledge regarding genetic control of physiological traits and the linkage of these physiological characteristics to molecular markers on chromosomes, and eventually the genes underlying the traits. Molecular markers have been rapidly adopted by researchers worldwide as an effective and appropriate tool for primary and practical studies addressing physiological traits. An important way of linking marker loci to a particular plant phenotype is through the use of genetic linkage maps (Graham et al., 2008).

### Genome-wide association mapping

In a large germplasm collection, an alternative QTL mapping approach is conducting association analysis, known as association mapping. In a population, association mapping is based on linkage disequilibrium (LD) or the non-independence of alleles. Association mapping studies using sparse SSR and SNP markers have been proven to be effective when identifying marker-trait associations in rice (Qiu et al., 2015; McCouch et al., 2016). Genome-wide association studies (GWASs) by means of high density markers have become increasingly popular in rice genetics with the development of high-throughput sequencing and SNP chip techniques (Abe et al., 2010; Yu et al., 2014). GWAS is a powerful strategy to clearly understand the genetic basis of complex traits that has been especially productive for rice (Qiu et al., 2015).

## Gene pyramiding using MAS: a smart approach to breeding programs

Gene pyramiding in rice is the transfer or pyramiding more than one resistance/tolerance genes/QTLs into a single rice genotype (Figure 1B) (Collard and Mackill, 2008). Pyramiding of resistance genes into a single line for each disease or stress is a novel strategy in rice breeding to prevent the breakdown of resistance against specific disease or stress. Pyramiding of genes/QTLs that confer resistance to biotic stresses and tolerance against various types of abiotic stresses is now feasible because of advancements in molecular markers (Das and Rao, 2015). MAS has been found to work efficiently for transferring genes from pyramided lines into new plants and into the improved varieties (Magar et al., 2014).

Breeders have used marker-assisted selection to “pyramid” resistance conferred by several separate resistance genes/QTLs with the help of closely linked markers against diseases such as BB, RB, and gall midge in rice, leaf rust resistance and powdery mildew resistance in wheat, and insect pest resistance in cotton, as well as for several abiotic stresses such as submergence, salinity, drought, and cold stress (Table 1) into rice, wheat, and cotton (Das and Rao, 2015; Pradhan et al., 2015; Suh et al., 2015; Shamsudin et al., 2016).

To get the desired population with required gene combinations without unwanted genes, backcrossing with the recurrent parent is required. The use of molecular markers which were unlinked to the assembled genes/QTLs, for back ground selection enhances the proportion of recovery of the recipient genome (Figures 1A,B). The gene pyramiding scheme can be distinguished into two parts, development of a pedigree, which is designed to accumulate all target genes in a single genotype known as the root genotype, and a fixation step, which is intended to fix the target genes into a homozygous state to derive the ideal genotype from one single genotype (Figure 1B).

## Prospects of molecular methods managing stresses affecting rice productivity

### Abiotic stress

Abiotic and biotic (Table 1, Figure 1C) stresses are the two major constraints accountable for the declination of growth and productivity of rice varieties (Wani and Sah, 2014). Abiotic stress (Table 1, Figure 1C) influence the distribution of plant species, survival rate, and the yields of the crop (Lee et al., 2005). Each year, about 25% of the rice croplands worldwide are submerged by flash floods (Loo et al., 2015). With a number of minor QTLs, submergence tolerance in rice is controlled by a single major QTL on chromosome 9 that provides complete submergence tolerance for up to 14 days or more (Septiningsih et al., 2009).

Sensitivity of rice to salt-stress changes throughout the lifecycle, but the effects are most severe in the seedling and reproductive stages (Thitisaksakul et al., 2015). Several salt-resistant rice varieties have been produced by expressing salt-responsive genes. QTLs associated with salt tolerant rice varieties can be mapped using microsatellite markers (Singh et al., 2007). Use of molecular breeding techniques have been shown to be the most efficient tools for development of improved varieties tolerable to salt (Mondal et al., 2013). In the salinity-tolerant cultivar NonaBokra, mapping of SKC1 on chromosome1 was a breakthrough that preserved K^+^ ion homeostasis under salinity conditions (Ren et al., 2005; Das et al., 2015).

Significant improvements have been made in mapping QTLs for drought resistance traits in rice; however, few have been effectively used in marker-assisted breeding (Prince et al., 2015). A number of drought tolerant QTLs for rice have been identified (Huang et al., 2014). DREB transcription factors play a major role in induction of the expression of genes involved in drought stress, and the genes encoding DREB transcription factors exhibit significant enhancement of the response of plants to drought stress (Udvardi et al., 2007). Several approaches have been improved to enable identification of low temperature stress resistant rice varieties. Cold tolerance of rice in the seedling stage is controlled by multiple genes and several QTLs (Zhang et al., 2014). In 2014, Xu and Cai reported that the *Ran* gene, *OsRAN1*, is necessary for the development of cold tolerant rice varieties. Pyramiding of cold resistance QTLs using MAS is useful for improvement of new rice cultivars with cold tolerance (Shinada et al., 2014).

### Biotic stress

Biotic stresses in rice (Table 1, Figure 1C) are caused by insects, including the gall midge and brown plant hopper, and by diseases including bacterial blight, blast, and sheath blight. Asian rice Gall-midge (GM), *Orseolia oryzae* (wood-mason), is a serious pest of rice in China, India, Sri Lanka, and several other countries (Katiyar et al., 2000). So far 11 GM resistance genes have been acknowledged in various rice varieties, *Gm1, Gm2, gm3, Gm4, Gm5, Gm6, Gm7, Gm8, Gm9, Gm10*, and *Gm11(t)* (Dutta et al., 2014; Das and Rao, 2015; Hasan et al., 2015; Bentur et al., 2016). The brown plant hopper (BPH), *Nilaparvata lugens*, has been one of the most devastating pests to rice crops in Vietnam and Asia. There is successful report of the use of SSR and STS markers in pyramiding two BPH resistance genes *Bph14* and *Bph15* into three elite japonica varieties Shengdao 15, Shengdao 16, Xudao 3 using marker assisted backcross breeding program (Xu, 2013).

Rice cultivation across tropical and semi-tropical regions of the world is affected by bacterial blight (BB) disease caused by *Xanthomonas oryzae* pv. *oryzae (Xoo)*. A total of 38 *R* genes of BB have been identified in rice (Khan et al., 2014). Resistant cultivars with one or two major resistant genes are unsustainable in the field and the only way to delay such a breakdown of BB resistance is to pyramid many resistance genes using MAS (Rafique et al., 2010).

Rice sheath blight disease caused by *Rhizoctonia solani* Kuhn reduces trivial yield in rice-growing areas around the globe (Yellareddygari et al., 2014; Yadav et al., 2015). Genetic studies have shown that SB resistance can be controlled by polygenic QTLs. Using MAS it is possible to pyramid SB resistance QTLs into rice varieties. Rice blast disease (RB), is generally considered as the most important rice disease worldwide (Divya, 2013; Miah et al., 2013). RB is caused by a filamentous heterothallic ascomycetous fungus, *Pyricularia grisea*, which is known as *Magnaporthe grisea* (Hebert) Barr. in its sexual state (Divya, 2013). Rice tungro (RT) disease consists of a spherical RNA virus (RTSV) and a DNA bacilliform virus (RTBV). The green leafhopper (GLH), *Nephotettix virescens*, is the most proficient vector of rice RT virus disease.

## Conclusions

There are several abiotic and biotic stresses that affect the productivity of rice cultivation throughout the world. To meet the demands of the growing population, there is urgent need to protect rice plants from various abiotic and biotic stresses that reduce yield and quality. Presently, the conventional breeding of rice is rapidly advancing due to the integration of molecular markers and MAS techniques. Through the application of molecular markers with the help of MAS in gene pyramiding, multiple stress resistant genes could be incorporated into a single rice variety with high yield, abiotic stress tolerance and biotic stress resistance with enhanced nutritional quality.

## Author contributions

GD and JP collected literature and wrote the manuscript. KB edited the manuscript.

### Conflict of interest statement

The authors declare that the research was conducted in the absence of any commercial or financial relationships that could be construed as a potential conflict of interest.
