# Corrigendum: Insight into MAS: A Molecular Tool for Development of Stress Resistant and Quality of Rice through Gene Stacking

**DOI:** 10.3389/fpls.2017.01321

**Published:** 2017-07-25

**Authors:** Gitishree Das, Jayanta Kumar Patra, Kwang-Hyun Baek

**Affiliations:** ^1^Research Institute of Biotechnology and Medical Converged Science, Dongguk University Seoul Goyang-si, South Korea; ^2^Department of Biotechnology, Yeungnam University Gyeongsan, South Korea

**Keywords:** gene pyramiding, genome mapping, phenotype traits, physiological traits, molecular markers, marker assisted selection, rice

In the original article, there was a mistake in **Table 1** as published. In the row 2 of the **Table 1** (Samba Mashuri BPT-5204), the reference was wrongly cited as “Kottapalli et al., 2010”. The corrected reference is “Sundaram et al., 2008.” The authors apologize for this error and state that this does not change the scientific conclusions of the article in any way.

## Conflict of interest statement

The authors declare that the research was conducted in the absence of any commercial or financial relationships that could be construed as a potential conflict of interest.
